# Developing Rat Bone Marrow Derived Mast Cells by the Splenic Cells Culture Supernatant of Rat and Mouse

**Published:** 2019-02

**Authors:** Saeede Amani, Rasoul Shahrooz, Ali Karimi, Zahra Bakhtiari, Esmaeil Mortaz

**Affiliations:** 1 Department of Comparative Histology and Anatomy Sciences, Faculty of Veterinary Medicine, Urmia University, Urmia, Iran,; 2 Clinical Tuberculosis and Epidemiology Research Center, National Research Institute of Tuberculosis and Lung Diseases (NRITLD), Shahid Beheshti University of Medical Sciences, Tehran, Iran,; 3 Department of Immunology, Faculty of Medicine, Shahid Beheshti University of Medical Sciences, Tehran, Iran.

**Keywords:** Bone marrow, Mast cell differentiation, Rat splenic cell culture

## Abstract

**Background:**

Mast cells play a critical role in the pathogenesis of various immunological and non-immunological diseases. It is now accepted that culturing primary mast cells considered as a tool for investigation role of mast cells in diseases. Development of various animal primary mast cells and their function could be used for the translational studies in the pathogenesis of human diseases. The aim of the study was to develop simple and cost-efficient method for differentiation and culture of rat mast cells from bone marrow by using rat and mouse spleen supernatant.

**Materials and Methods:**

Bone marrow cells from 10 to15-weeks-old male rats was obtained and cultured for three weeks on cell culture medium. After that, purity of cells was approved by FCɛRI and CD117 antibodies, toluidine blue and Immunohistochemistry (IHC).

**Results:**

After 3 weeks continuous culturing, high purity of cells was found. CD117, CD34 expression and tryptase were 80.1, 76.89 and 87.9%, respectively by rat splenic supernatant, whereas 85.4, 83.07 and 82.1%, respectively with mouse splenic supernatants. Besides, rat spleen supernatant developed 91.4% and mouse splenocyte supernatant developed 89.7% mast cells based on surface markers.

**Conclusion:**

The data presented in this study indicated equal maturation and differentiation of bone marrow derived rat mast cells by using both spleen supernatants.

## INTRODUCTION

Mast Cells (MC) are paracrine cells playing important role in allergic and non-allergic reactions (1). These cells contain receptors with high affinity for immunoglobulin E (IgE) (1, 2). Due to difficulty in isolating high numbers of mast cells from animals, cell lines are commonly used for biological and functional investigations (3). For example, the biology of mast cells has been limited to a species of mast cells known as connective tissue mast cells, as they could be easily obtained, especially from the peritoneal cavity. Moreover, study of mucosal mast cells and basophils have been neglected due to difficulties in their isolation (4). Emerging data, identified factors controlling the growth and differentiation of human mast cells. Stem Cell Factor (SCF) and IL-3 were considered as a source of stimulator of mast cells in in-vitro many years ago (5). However, IL-3 does not seem to be a growth and differentiation factor for (normal) human mast cells in bone marrow or peripheral blood cells culture (4). CD117 receptor which is specific for mast cells binds SCF and accounts for maturation and differentiation of mast cells.

IL-3 is the major differentiating factor for basophils and others inflammatory cells (6). Recently, human mast cells growth was successfully induced in long-term cultures of umbilical cord blood cells by a fibroblastic cell line (4). Mast cells can be generated from different sources for *in vitro* studies (7). These cells nowadays are considered for the studies of reconstructive investigations (8). Studies performed on mast cells have been done mostly on BALBc mice (9). There is few exceptions in mast cells of rat and mice for example proliferation, quantity of metachromatic granules (10, 11), and life span. Important issue is life span of rat mast cells, which is shorter (5 weeks) than the mice (8–12 weeks) (11, 12). Finding the differences of mast cells of two species is important in mast cell biology investigation. Thus, the current study aimed to characterize the isolation protocols of these cells by using two kinds of stimulations for proliferation and differentiations of cells i.e. spleen supernatant culture from mouse or rat sources. In this way femur isolated bone marrow cells were cultured in two different conditions with rat splenic cells supernatants or mouse splenic cell supernatants origin for 3 weeks. After 3 weeks the purity and specific markers of cells were evaluated by Immunohistochemistry (IHC) and flow cytometry analysis.

## MATERIALS AND METHODS

### Bone Marrow cell isolation

The study was carried out based on the International Association for the Study of Pain (IASP) (13) and research board of Urmia University which approved all the experiments sets. All rats were first anesthetized by intraperitoneal Ketamine-Xylazine (ketamine 5%, 90 mg/kg and xylazine 2%, 5 mg/kg) and then euthanized by a high-dose of CO_2_ (2).

Bone marrow cells were immediately isolated from rat femur and tibia bones as described earlier (14). Then the bones were flushed by insulin syringe using endotoxin-free culture medium and obtained materials were centrifuged for 10 minutes at 320xg at 4°C. Then cells were cultured at the ratio of 0.5×10^6^/ml in complete media (RPMI1640 containing FBS 10%, 100 IU/ml Penicillin, 100 μg/ml Streptomycin, 0.1 μmol of non-essential aminoacids, 2 mmol L-glutamine) and splenic mitogen pokeweed (20%). The medium was changed every 5 days. After 3–4 weeks, cells were washed with cold PBS (1X) and then evaluated as described in next part.

### Pokeweed mitogen-stimulated spleen cell conditioned medium

Splenic cells were isolated from rat and cultured with density of two million cells/ml in RPMI1640 medium which contained 4 μmol of L-glutamine, 5×10^−5^M 2-mercaptoethanol, 1 mmol Sodium pyruvate, 100 IU/ml Penicillin, 100 μg/ml Streptomycin, 0.1 μmol non-essential amino acids, and 8 μg/ml Lectin in 75 cm^2^ flasks. After 5–7 days the supernatant culture medium was centrifuged for 15 minutes at 3200g and was passed through 0.22 μm filter, and the obtained fluid was used as pokeweed splenic mitogen (15).

### Toluidine blue metachromatic stain for mast cells

Toluidine blue staining used was as follows: **a)** Fixation of cells with paraformaldehyde, 3–4% neutral, **b)** Pouring the toluidine blue solution on the fixed slides which contains toluidine blue 0/1 mg and distilled water 100.0 ml) for 1–2 min. Then the slides were covered by coverslip using finger nail polish (16).

### ICC of CD34 and CD117

The cells are placed on a slide and were fixed with paraformaldehyde 4% and then slides were washed with PBS for 10 minutes and entered to the specific staining stage afterward. Immunocytochemistry staining steps were performed according to the protocol to the manufacturer instructor (Novocastra, U.K.). These steps could be summarized as follows:

- Flooding the slides in peroxidase black for 5 minutes,
- Flooding the slides in protein blocker for 5 minutes,
- Flooding the slides in post-primary for 30 minutes,
- Flooding the samples in Novolink polymer for 30 minutes,
- Flooding slides in DAB chromogen for 10–15 minutes.

Then the slides were flooded in PBS for 5 minutes between each two steps. Then the cells were counterstained by Gill’s II hematoxylin (Fisher Scientific, Fair Lawn, NJ,) and fixed using crystal/mount (Biomeda, Foster City, CA, USA) and prepared for the study (17) .

### ICC (Immunocytochemistry) of Tryptase

The cells were placed on a slide and fixed with paraformaldehyde 4%. Then the slides were washed in PBS for 10 minutes and later enter the specific staining stage. Slides were immersed in sodium citrate buffer to retrieve antigen, pH 6.0, in an 86 °C water bath for 15 min. After being rinsed with water, protocols were followed by incubation in PBS for 5 min, and then slides were blocked with normal goat serum (Vector, Peterborough, UK) for 20 min. After that the slides were incubated with 150 μl of rabbit anti-mouse tryptase (mouse mast cell protease-6) (Santa Cruz, Tebu-Bio, The Netherlands) primary antibody in predetermined optimal dilutions for 1 hour at room temperature, washed in PBS, and incubated for 30 min in 500 μl of goat anti-rabbit biotinylated secondary antibody (DAKO, Denmark) diluted 1:150 in PBS. Hereafter, slides were washed in PBS and incubated in freshly complexed avidin–biotin alkaline phosphatase ABC reagent (Vectastain ABC kit; Vector) for 20 min. After a final series of washes in PBS, 200 μl of fast red [(1 mg/ml) (Sigma-Aldrich, The Netherlands)] substrate was applied for 25 min. This reaction was stopped by rinsing with distilled water, and the tissue was subsequently counterstained with Gill's II hematoxylin (Fisher Scientific, Fair Lawn, NJ) and mounted with Crystal/Mount (Biomeda, Foster City, CA) as described earlier (18).

### Characterization of mast cells by flow cytometry

Harvested cells (about 50000 cells) were incubated with cold PBS before staining with Abs, and then cells incubated with the cell-surface Fc blocker receptors antibody (PharMingen, San Diego, CA, USA) for 15 min. Then after centrifugation the cells were incubated with PE-conjugated anti-rat *c-kit,* (c-Kit (2B8): sc-19619, USA)*,* and FITC-conjugated anti-rat Fc*ɛ*RI antibody (BD Pharmingen: No.551469, USA) for 30 minutes at 4°C. In parallel isotype-matched non-specific Abs were used as the background staining control. Live events were acquired on a FACSCalibour flow cytometer (BD Biosciences), and data were analyzed with FACSDiva software (v6.1.2) (19).

## RESULTS

**Figure 1.**
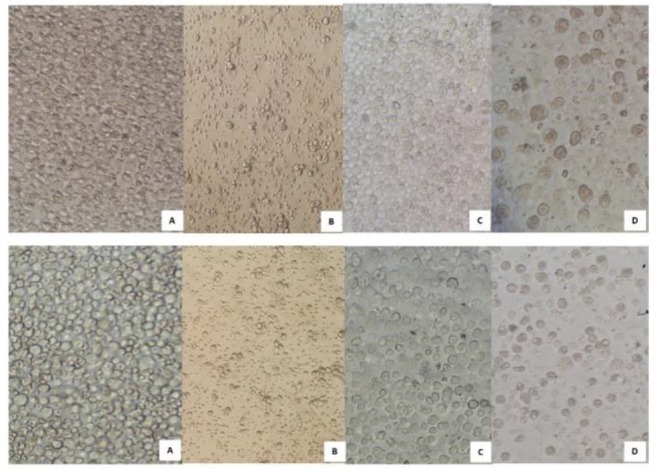
Bone marrow cells culture on the first day (A). Heterogeneous isolated cells on the fifth day (B). Heterogeneous isolated cells on the tenth day (C). Homogeneous mast cells on day 23(D). With splenic supernatant of rat (upper panel) and mouse (lower panel).

In the first culture of the bone marrow cells, both adhesive and non-adhesive cells were grown with heterogeneous format (Figure 1A). On the days 8–12 of culture, after third or fourth passages, the number of cells reduced dramatically; then the bullish trend growth of cells were increased (1B and C). Moreover, culturing of cells in density of 3× 10^5^-1 × 10^6^ cells/ml resulted in high yield. The floating cells, 18 and 19 days after the fourth culture gave rise to condensation with more homogeneous appearance. After 4 to 5 passages, on day 23, the cell morphology seems in mature format containing granules and round shape (Figure 1 D). Additionally, we examined splenic cell culture supernatant of mouse for the rat mast cell of isolated cells. No differences between two mast cells were observed with both splenic supernatants.

**Figure 2.**
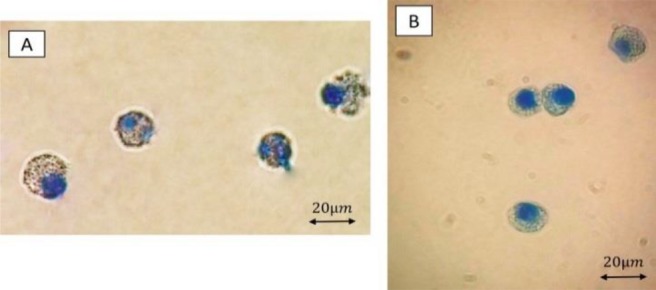
Metachromatic (red-purple) granules of mast cells of Rat (A) and Mouse (B) which have clearly filled the mast cells’ cytoplasm. Toluidine blue staining of cells at magnification of 1000X.

The harvested cells were then counted and their viability was evaluated using Trypan Blue (TB) exploration stains. 12,000,000 cells with the viability of 90% (detected by TB staining) were harvested from each flask. After harvesting the cells with distinct splenic supernatant and their staining with toluidine blue, mast cell granules stained purple to red in two mature mast cell crowd (Figure 2).

### Immunocytochemical analysis of mast cells

**Figure 3.**
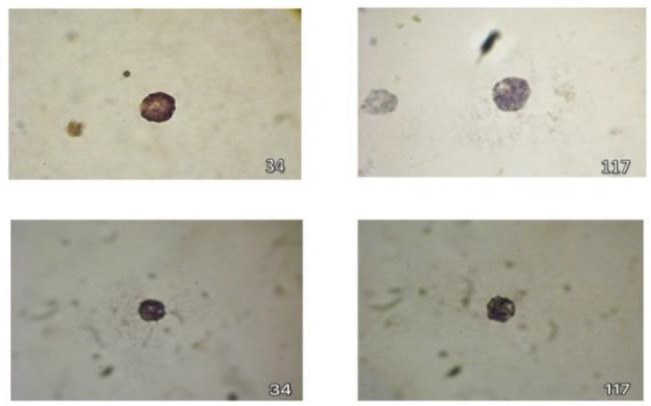
The reaction of CD117 was identified with dark stain and CD34 with light brown stain. Immunohistochemistry staining of CD117 and CD34 by rat (upper panel) and mouse (lower panel) splenic supernatants. 1000X magnification

**Figure 4.**
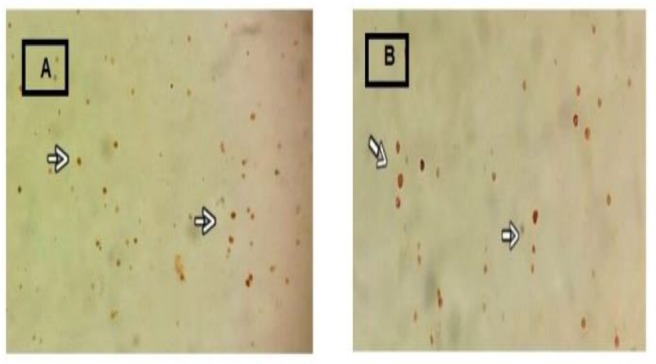
Tryptase IHC in the mast cell cytoplasm was identified by brown colour. Immunohistochemistry staining for tryptase enzyme by rat (A) and mouse (B) splenic supernatant. 400X magnification

In the next step, specific markers for differentiated bone marrow stromal stem cells, including CD117 (c-kit), CD34, and tryptase were analyzed. CD117, CD34 markers and tryptase were positive 80.1, 76.89 and 87.9%, respectively, with rat splenic supernatant, whereas were positive 85.4, 83.07 and 82.1%, respectively with mouse splenic supernatants (Figures 3 and 4). All the three markers played an important role in confirming the specific characteristics of mast cells.

**Figure 5.**
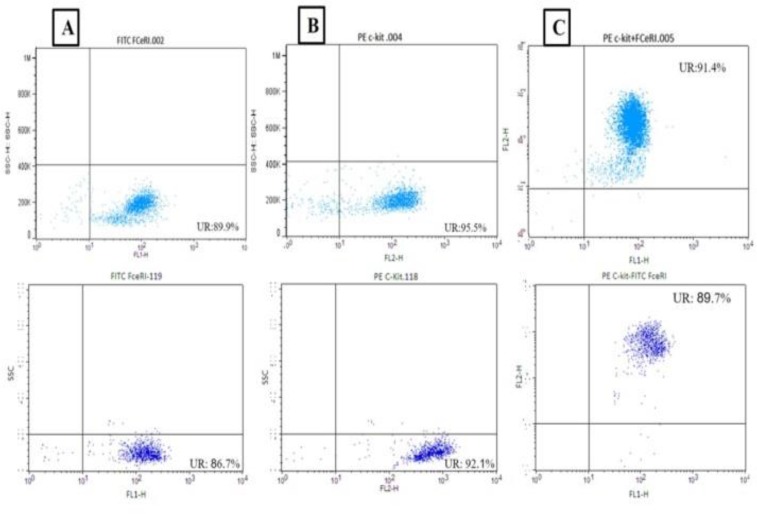
Flow cytometry analysis of rat bone marrow-derived mast cells (BMMC). A) Positive cells for FCϵRI, B) Positive cells for CD117 (c-kit), C) Double positive cells; upper panel indicated for cells cultured with rat supernatant of spleen (91.4%) and lower panel stand for rat mast cells with mouse splenocyte supernatant (89.7%).

**Figure 6.**
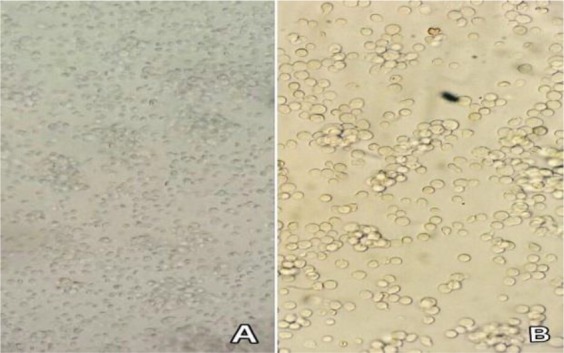
Obtaining pokeweed (splenic supernatant) from spleen:

ICC result is confirmed by flow cytometry analysis of rat and mouse spleen supernatant growth of bone marrow-derived MCs. Rat spleen supernatant developed 91.4% and mouse splenocyte supernatant developed 89.7% mast cells based on double positive for FCɛRI and CD117 surface markers (Figure 5). Furthermore, splenic cell culture supernatant (POKEWEED) was used to stimulate and activate mast cells. We examined splenic cell culture supernatant of mouse for the rat bone marrow isolated cells (Figure 6).

Homogenized splenic cells which contain culture medium on the first day.

Splenic cell and culture medium on day 7 (6).

## DISCUSSION

MCs have been described as important effector cells in the immune system response. MCs are widely distributed in all the tissues of body (11). Developing primary mast cells with high yield in in-vitro protocols, is considered important in studies of mast cells function. For these reasons, few investigations try to introduce appropriate procedure to generate large numbers of homogenous non-transformed mast cells. However, there are many limitations for presenting cellular model human MCs due to their low number and high price techniques. In this line usage cell lines is much appreciated (11, 14). But still, need to develop primary cells to dissect the molecular pathways that are required for MC differentiation and specific biological activities.

Compared to other myeloid cells, not much information for the mast cell biology is available, which could be due to be their low number (0.002–0.05%) and difficult in isolation procedures (20). Although sampling of bone marrow cells is a prominent method for obtaining mast cells, its sensitivity and accuracy are limited due to the heterogeneous origin and a low yield of mast cells. However, by introduction of accurate methods, the mast cells for *in-vitro* studies could be obtained from a variety of tissues (7, 21), such as Bone Marrow-derived Mast Cells (BMMCs) (22), skin-derived mast cells (FSMCs) (7), and peritoneal-derived BMMCs which may be used in in vitro studies. Due to the large yield of generated mast cells from animals it is possible to use in knock out animal for reconstitution studies (23). Moreover, some mast cell lines are not responsive to allergic and non-allergic stimuli as well as mast cell knockout mice (24).

In the current study, generation of rat mast cell by culturing bone marrow cells with spleen supernatants (obtained by rat and mice) was developed after 3 weeks. The cytomorphology of harvested rat MCs in supernatants of the mouse spleen cells was similar to the mouse mast cells. The main histochemical difference of mast cells is their differentiation in metachromatic staining indicating the difference in proteoglycan contents of mast cells cytoplasmic granules (24). Toluidine blue has been widely used to determine the granules of mast cells (25). Polychromatic stain could exhibit different colours based on its chemical attachment to different tissue components. The main histochemical difference of mast cells relative to other cells, especially the basophils, is its differentiation in metachromatic staining (24, 26). These granules are purple because of heparin and histamine contents (26).

Mast cells tryptase and CD117 (14) considered as important markers of cells. Tryptase –a neutral protease- is a specific marker for mast cells which is stored exclusively in secretory granules of mast cells (20, 27). Tryptase accumulates in the secretory granules of mast cells, not basophils, and released along with histamine and acts like a diagnostic evidence (26).

Staining of CD117 and SCF important in mast cells characterization since these markers have been shown in melanocytes, intestinal cells, some stem cells, and earliest lymphoid progenitors (14). Besides, CD34 is a 90–120 kDa superficial sialo-mucin molecules which has been used as a diagnostic factor in hematopoietic stem cells (HSCs), mast cells (mature form) (27), and vascular endothelial cells(28), whereas, human mature mast cells do not express CD34 on the surface (28).

New protocol of culturing rat mast cells by mouse spleen supernatant indicated the identical ratio of development and differentiation with rat spleen supernatants. It has been shown that the number of mast cells by bone marrow cells depends on their progenitors production rate and viability of mature mast cells in tissues (29). Mast cells are activated through the stimulants such as cross-linking IgE, and SCF (30). Activation and differentiation of mast cells are regulated by IL-3 and SCF, respectively (31). C-kit, CD117, and SCF receptors are important factors for survival, differentiation, and maturation of MCs (29, 32). Although SCF is the essential factor for mast cells survival, FCƐRI can also improve their viability (30). In the rat, IL-3 is known as a mast cell growth factor (33), thus mast cells are generally prepared from bone marrow cells using culture with IL-3 or a supernatant containing IL-3 (34). Recent reports have demonstrated that SCF is another c-kit ligand as mast cell growth factor in splenic cells culture (35). Several types of factors have been identified which can affect progenitor hematopoietic cells for the lineage differentiation. A suspension pokeweed with stimulatory mitogens of splenic cells produces medium condition and is able to stimulate ancestral cells of granulocyte-macrophage, eosinophils, megakaryocytes colony, and mast cells generation and differentiation (36). CD4+ T lymphocytes produce a significant amount of IL-3 in spleen supernatants. SCF is produced by fibroblasts in laboratory conditions (37). These cells exist in the splenic connective tissue and produce SCF and IL-3 in in-vitro conditions for survival and spread of mast cells (7). Thus, it has been concluded that rat mast cells development by mouse spleen supernatants is the same as rat spleen supernatants. Finally, here, we propose a practical and inexpensive method for purifying rat mast cells and conditions for generating pure cultured rat mast cell from bone marrow.
